# How do physicians and trainers experience outcome-based education in “Rational prescribing”?

**DOI:** 10.1186/1756-0500-7-944

**Published:** 2014-12-23

**Authors:** Hamideh M Esmaily, Rezagoli Vahidi, Niaz Mousavian Fathi, Rolf Wahlström

**Affiliations:** Medical Management Centre (MMC), Department of Learning, Informatics, Management and Ethics (LIME), Karolinska Institutet, Stockholm, Sweden; Educational Development Centre (EDC), Tabriz University of Medical Sciences, Tabriz, Iran; National Public Health Management Centre (NPMC), Tabriz, Iran; Department of Health and Nutrition, Tabriz University of Medical Sciences, Tabriz, Iran; Department of Pharmacology & Toxicology, Tabriz University of Medical Sciences, Tabriz, Iran; Department of Public Health Sciences, Karolinska Institutet, Stockholm, Sweden

**Keywords:** Continuing medical education, Primary care, Outcome-based education, Educational intervention, Rational prescribing, Experience, Perception, Educational planning, Effectiveness

## Abstract

**Background:**

Continuing medical education (CME) is compulsory in Iran, but has shown limitations in terms of educational style and format. Outcome-based education (OBE) has been proposed internationally to create links to physicians’ actual practices. We designed an outcome-based educational intervention for general physicians in primary care (GPs). Positive outcomes on GPs’ knowledge, skills and performance in the field of rational prescribing were found and have been reported.

The specific purpose of this study was to explore the perceptions of the GPs and trainers, who participated in the outcome-based education on rational prescribing.

**Methods:**

All nine trainers in the educational programme and 12 general physicians (out of 58) were invited to individual interviews four months after participation in the CME program. Semi-structured open-ended interviews were carried out. Qualitative content analysis was used to explore the text and to interpret meaning and intention.

**Results:**

There was a widespread agreement that the programme improved the participants’ knowledge and skills to a higher extent than previously attended programmes. Trainers emphasized the effect of outcome-based education on their educational planning, teaching and assessment methods, while the general physicians’ challenges were how to adapt their learning in the real work environment considering social and economical barriers. Self-described attitudes and reported practice changed towards more rational prescribing.

**Conclusions:**

Outcome-based CME seems attractive and additionally useful for general physicians in Iran and could be an effective approach when creating CME programmes to improve general physicians’ performance. Similar approaches could be considered in other contexts both regionally and globally.

## Background

Continuing Medical Education (CME) will continue to play a crucial role in improving health care professionals’ performance all over the world in the 21^st^ century [1–3]. The actual learning needs of practitioners are usually not met within traditional CME programmes, although physicians’ knowledge may increase [4–7]. However, the challenge is to bridge the gap between health professionals’ knowledge and their everyday practice [8, 9]. The effectiveness of CME activities should therefore be evaluated at the highest possible level, which means outcomes at practice level, either the physician’s performance or patients’ health outcomes [10, 11].

Outcome Based Education (OBE) is an educational approach emphasizing the continuum of education from undergraduate education to CME [12, 13]. OBE can influence the whole process of education from decisions about the content of the curriculum; formulation of aims; the educational strategies; design of teaching methods; assessment procedures; and the educational environment [12, 14]. Some studies conducted in Canada, USA and the UK have presented OBE as an innovative, interactive and effective approach when planning for CME [15–17]. So far, we have found no such studies reported from Asia.

Rational prescribing by doctors is of high importance since inappropriate prescribing behaviour may lead to unsafe treatment [18, 19]. Several studies have indicated overprescribing, multi-drug prescribing, and overuse of antibiotics, injections and NSAIDs as common problems of irrational drug use in different countries [20–23] as well as in Iran [24–27]. According to Quick et al. [28, 29], there are four types of intervention strategies to improve drug use: (i) educational; (ii) managerial; (iii) financial; and (iv) regulatory. Educational interventions are mostly used for prescribers and consumers of services.

This study was part of a larger project with the aim to assess if OBE was effective, useful and appropriate in CME programmes for general physicians in primary care (GPs), through an intervention in the field of “Rational prescribing” [30–32]. An outcome-based approach had not been used previously in CME programmes in Iran.

### Summary of the intervention project

The project was designed as a cluster randomized controlled trial (CRCT). Firstly, 21 learning outcomes were identified through a modified Delphi process. The OBE indicators were used by expert panels to determine six educational topics for the CME programme and define the curricular content for each topic [30]. The six topics were 1) Principles of prescription writing, 2) Adverse drug reactions, 3) Drug interactions, 4) Injections, 5) Antibiotic therapy, and 6) Anti-inflammatory agents therapy. All GPs working in six cities in the East Azerbaijan province in Iran were invited to participate in the educational programme. The cities were matched and randomly divided into an intervention arm for education within an OBE programme, and a control arm for a traditional CME programme. Knowledge and skills of participants were assessed using a pre- and post-test and their prescribing behaviour was assessed through collecting 10% of their prescriptions, nine months before, and three months after the programmes.

In total, 112 GPs out of 159 participated in the programme. There were significant improvements in knowledge and prescribing skills after the training in the intervention arm as well as in comparison with the changes in the control arm [31]. The GPs in the intervention arm significantly reduced the total number of prescribed drugs and the number of injections per prescription. They increased their compliance with specific requirements for a correct prescription, such as explanation of specific time and manner of intake and precautions necessary when using drugs [32]. However, compared with the control arm, there was no significant improvement regarding prescribing antibiotics and anti-inflammatory agents.

The specific purpose of this study was to explore the experiences and perceptions of the GPs and the trainers regarding the usefulness and effectiveness of this new outcome-based educational approach, in relation to the GPs’ prescribing practices and the trainers’ ability to create an appropriate learning environment.

## Method

This was a qualitative study with individual interviews.

### Study setting

The main CRCT study was conducted in three cities in the Northern part of the East Azerbaijan province in Iran. Seventy-four GPs had been invited to participate in the CRTC mentioned previously and 58 accepted to participate in the outcome-based educational intervention programme, which was led by nine specifically trained educators. The participants in this qualitative study were selected from this group of GPs and trainers.

### Participants

#### Trainees

Seventeen GPs who had participated in the programme were purposively selected based on variation in age, gender, years in practice and city of practice, and invited by the first author (HME) to individual interviews, four months after participating in the CME programme.

#### Trainers

All nine trainers of the OBE programme (2 women, 7 men; 5 medical specialists, 4 pharmacists) were invited to an interview five months after participating in a teacher training workshop on OBE, which took place about one month before their teaching in the CME programme. All of them were faculty members of Tabriz University of Medical Sciences and experienced CME trainers.

#### Interviews

Semi-structured guides (list below) were used for the interviews, which were conducted during January-February 2007. All interviews were recorded on audiotape with permission of the interviewees. The length of the interviews was 30–40 minutes with GPs, and 40–60 minute with trainers. Content validity of the interview guides was verified by experts in the medical education field. The first author conducted all interviews. The venue and time of the interview were selected by each participant. The interviews with the GPs were conducted in the local language (Azeri), and were later translated into Farsi, whereas the trainers were interviewed in Farsi. Thereafter all interviews were translated verbatim into English. Two of the Iranian research team members verified the accuracy of the translations.

### The interview framework for trainees of outcome-based education

-  Have you participated in any CME programmes before the outcome-based one?
-  Have you participated in any rational prescribing CME programme before the outcome-based one?
-  Tell me about the outcome-based programme you participated in; (content, trainers, teaching methods, your own contribution during educational programme, educational materials you received, evaluation methods.
-  Can you describe if there has been any changes in your:
  - Knowledge in discussed subjects
  - Attitude about prescribing
  - Prescribing skills- PerformanceIs there anything else you would like to mention?

### The interview framework for trainers of outcome-based education

-  Have you taught in any CME programmes before this outcome-based one?
-  Can I ask your point of view regarding outcome-based education?
-  Did you have the same educational strategies and methods in this outcome-based teaching as you usually had in other CME programmes?
-  Did the participants of outcome-based programme behave as usual as in other CME programmes?
-  Do you have any comments about outcome-based education? Is there anything else you would like to mention?

The GPs were asked to describe their experiences related to the recent educational programme in order to explore what aspects of OBE that might be effective with respect to improving their knowledge, attitudes, skills and performance (list above). The trainers were encouraged to explain their point of view regarding this approach and to describe their educational strategies and methods. Trainers were also asked to explain how involved the participants were in comparison to other CME programmes (list above). Data saturation for GPs was reached after twelve interviews (5 women, 7 men), as no new information emerged from the data, and no further interviews were conducted. All nine trainers were interviewed.

### Analysis

Qualitative content analysis [33, 34] was used for analysis of the transcribed data considering what the transcripts comprised, and through interpretation of meaning and intention [35, 36].

Firstly, the interviews were read and re-read by the first author to capture a sense of the whole. Then the meaning units were identified and suitable codes were attached. One member of the research team (LOD, see Acknowledgment) re-coded some of the texts and meaning units, serving as an inter-rater and thus increasing the reliability of the codes. The themes discerned through the analysis and meaning units and codes were reduced to appropriate sub-themes under five main themes, which were checked with the other authors until consensus was reached [37].

For trustworthiness, the principal investigator did member, peer and expert check. In this regard, transcripts were checked with participants and all meaning units were checked by two researchers outside the team.

Direct quotes were provided to illustrate and exemplify the themes as well as the sub-themes. These were selected based on their relevance for the sub-themes and themes to give the reader an opportunity to assess the feasibility of the themes and sub-themes suggested by the authors. The sources of the quotes have been abbreviated as “P” with a number for the GPs as participants in the CRCT, and “T” with number for the trainers.

### Ethical considerations

Ethical approval for the study was received from the National Ethics Committee of the Iranian Ministry of Health and Medical Education in 2005. All trainers and GPs, who were invited to an interview, were informed that their identity was protected and that their answers would be confidentially handled. Their willingness to participate was secured and their informed consent was obtained.

## Results

We created one set of themes and sub-themes for the GPs and one for the Trainers, respectively (Tables 1, 2).

**Table 1:** **Themes and sub-themes for GPs**

	GPs
Theme	Sub-themes
1. Value of the programme	- Value for learning
	- Relevance to future practice
	- Benefits of peer education
2. Robust content and process features of the programme	- Relevancy of the content
	- New learning approaches
	- Provision of useful booklets
	- Opportunity for self-assessment
3. Higher motivation for continued learning	- Motivation to read and continue education
	- Trainers’ motivating role
4. Positive impact of the programme	- Positive behaviour changes
	- Improvement of quality of prescribing
	- Need to change attitude towards prescribing
	- Willing to engage in patient education
5. Barriers for application	- Irrational performance of other health professionals
	- Illogical requests of patients and risk of losing clients
	- Lack of time

**Table 2:** **Themes and sub-themes for trainers**

	Trainers
Theme	Sub-themes
1. Robust and clear outcomes	- Positive attitude towards identified outcomes
	- Comprehensive content of the programme
2. Strong adult learning approach	- Clear educational strategy
	- Benefits of the teacher training programme
	- Time limitation of the workshop
	- Participants’ active involvement
3. Importance of assessment	- Suitable assessment tools
	- Impact of the programme
	- Barriers for effectiveness of education on behaviour change
4. Interprofessional teaching	

For the GPs, our analysis resulted in description of five themes: 1) Value of the programme; 2) Robust content and process features of the programme; 3) Higher motivation for continued learning; 4) Positive impact of the programme; and 5) Barriers for application.

For the trainers, we found four themes: 1) Robust and clear outcomes; 2) Strong adult learning approach; 3) Importance of assessment; and 4) Inter-professional teaching.

The following sections describe each theme, separately for GPs and trainers.

### GPs

All but one of the GPs had participated in several CME programmes during their professional careers. One third of them had participated in at least one CME programme on ‘Rational prescribing’ before the outcome-based programme in this study.

### Theme 1: Value of the programme

#### Value for learning

The participants expressed that the programme was useful for them in several aspects, and mentioned that the programme stood up to their expectations. This kind of programme was regarded to be cost effective and oriented towards health outcomes. They found the programme to be a great opportunity to learn regarding all included topics. There was, however, some dissatisfaction about the antibiotic therapy session compared with the other topics of the programme. Some GPs mentioned that the hierarchy of topics was very rational as learning each topic was useful to better understand the next one.

*“I learned lots of scientific and practical issues about prescription writing during this programme despite 15 years of work experience. Most of the other CME programmes, which I have participated in, are not adapted to GPs’ professional needs. Some of them are specialised. Some of them are very primitive. Doctors participate in those programmes, only to receive CME points…”* (P3)

#### Relevance to future practice

Interviewed GPs emphasised the practical use of this programme in comparison with other CME programmes that they had experienced. There was a conviction that the knowledge acquired would be useful for improving everyday clinical work, in relation to prescribing of medications.

*“After long time since participating in the programme, still I remember my learned issues. When I start writing prescriptions, relevant subjects are repeated in my mind, so I consider them carefully as much as possible.”* (P4)

#### Benefits of peer education

Interviewees said that during the programme they had good opportunities to learn from each other. Several group discussions during different sessions had let them share their knowledge and refer to real experiences.

*“We were sitting around the table and were discussing different practical cases face to face…in this case we need to push ourselves to get involved in the discussions…I was trying to learn as much as possible and force my mind to use my previous knowledge to be able to show off what I had known and what I had learned.”* (P4)

### Theme 2: Robust content and process features of the programme

#### Relevancy of the content

Participants described that the content of the outcome-based programme was different than previously attended CME programmes. Receiving information of high relevance to their professional needs and information about new drugs in the market rather than repetition of something they studied at university was highly appreciated. According to the participants, trainers were experts in their scientific area and they received lots of new information during the sessions.

*“I had red three books relevant to prescribing before participating in this programme…I like to read books and apply for distant CME programmes instead of sitting in courses, but I found the content of this programme refreshing and useful … so sometimes it is good to directly participate in a programme.”* (P2)

#### New learning approaches

It was mentioned in several ways that the trainers’ method of teaching was different from previous CME programmes. Collaboration between trainers from different disciplines (medical doctors and pharmacists) during the learning sessions was a new experience for the GPs. They also said that the trainers had followed a logical structure from entirety to details and from theory to practice. Round-table arrangement that promoted close interaction with the trainers and other participants, and feeling comfortable to ask questions was appreciated by the GPs. They enjoyed listening to the trainers and to participate in group discussions despite the length of the programme.

#### Provision of useful booklets

Participants mentioned their surprise, when they for the first time after participation in a CME programme received two booklets one month after the end of the programme. They mentioned two main reasons for the importance of receiving the booklets. First, they were relevant and proper, and second, they contributed to a feeling of importance. They were also satisfied with booklets they received during the programme.

#### Opportunity for self-assessment

The GPs appreciated that for the first time in CME programmes there were tests that created an opportunity for the participants to make self-assessments.

*“Usually when participating in CME programmes, we go and listen to some lectures or don’t listen, then after finishing the programme we receive the certificate and leave. In this programme in the beginning we received a questionnaire about Rational prescribing … Honestly, first I didn’t want to answer any of the questions because I was not sure about many of them, but when I noticed that the questionnaire was anonymous, I did what I was supposed to. After a while, I received the same questionnaire in my office and when I looked at it, I knew more than 90% of the answers. I saw the improvement and I was so happy. It was very good that I could assess my knowledge.”* (P7)

### Theme 3: Higher motivation for continued learning

#### Motivation to read and continue education

The interviewees explained that after participating in the programme, their motivation for reading and learning increased, not only about the main subjects but also regarding other relevant topics, which they regarded as important issues for rational prescribing.

#### Trainers’ motivating role

According to the participants the trainers had a great role to motivate them for learning more and using what they had learned in practice, by asking relevant questions about their practice and preparing group discussions.

### Theme 4: Positive impact of the programme

#### Positive behaviour changes

Some of the GPs stated that they had changed behaviour and strongly asked for assessment of their prescriptions to find out how much they had improved. They mentioned that they had recognised their own irrational performance and that they had changed behaviour after the programme through considering principles of prescription writing. Most of them believed that they had decreased the number of drugs per prescription and reduced the amount of injections.

#### Improvement of quality of prescribing

When describing practice after the programme, some of the GPs emphasised the influence of the OBE on the quality of their prescriptions and their carefulness about drug interactions and polypharmacy when prescribing. Some of them believed that they were thoughtful about rational prescribing also before enrolment in the programme, but that they now were even more encouraged to practice according to their beliefs. The GPs felt satisfaction and pride in knowing that they were now writing correct prescriptions and also that they wanted to improve even more.

#### Need to change attitude towards prescribing

The GPs repeatedly mentioned that they and their colleagues needed to change their attitude to rational prescribing.

*“Most of the time we prescribe unnecessary drugs just for satisfaction of patients…we don’t want to lose our patients…my attitude changed about prescribing. I wish all colleagues…I mean both GPs and specialists to participate in this kind of programmes to change their attitude..”* (P9)

Nevertheless, some said that this kind of programme could change their attitude but not necessarily the performance, due to lots of external barriers.

*“It is very good to participate in this kind of programmes and I am sure the programme changed the attitude of most of colleagues regarding prescribing but it is not possible to fly by one wing. The health system should be changed to consider doctors’ economical situation.”* (P6)

#### Willing to engage in patient education

Attempts to change their patients’ attitude as an essential issue regarding rational prescribing was stressed in some interviews, as well as the importance of patient education despite difficulties in convincing them to withdraw irrational requests.

“… *one day I had a patient, my diagnosis was viral cold. When I explained to him about the medicines which I prescribed, he told me, what about Penicillin, didn’t you write penicillin for me, if I don’t take penicillin I never recover. I remembered the discussions we had on workshops about patient education and tried to explain to him that for viral diseases not only antibiotics are not useful but they also create some problems in the future. I talked a bit about antibiotic resistance… apparently he accepted and left my office. I don’t know if he went to another doctor to receive antibiotics or not but I was satisfied with making the right decision.”* (P1)

The GPs stressed the role of mass media to inform people concerning rational use of drugs and danger of self-treatment and necessity of this education to enhance people’s awareness.

### Theme 5: Barriers for application

#### Irrational performance of other health professionals

Most participants believed that the pharmacists and some other doctors have a large role in shaping irrational use of drugs. Pharmacists give drugs to patients without a prescription, and doctors follow patients’ irrational requests and sometimes prescribe totally incorrect drugs without considering their side effects.

*“…for example a patient comes to my office. Before referral he has bought and taken lots of unrelated drugs, what can I do, I am not magician, so I continue the irrational treatment. For simple infections I prescribe very strong antibiotics, I know this is irrational, but I am a follower and not a problem maker…”* (P10)

#### Illogical requests of patients and risk of losing clients

It was clear that some doctors were suffering from feeling forced to accept patients’ requests in order not to lose them as clients. They mentioned the difficulties they found when saying “no” to their patients’ irrational and sometimes harmful requirements. Some also expressed insecurity if they did not follow the patients’ or their guardians’ irrational requests and even that they might face physically violent behaviour.

*“…in the area that I am working, there is a belief that betamethasone is an antipyretic. One time I was physically attacked and beaten by the father of a child, because the day before he asked me to prescribe betamethasone injection for the poor child because of his fever. I didn’t accept and prescribed acetaminophen beside some other drugs based on my diagnosis. The next day they came back to the health centre where I am working. The child had a convulsion … and the stupid father had thought the reason was lack of betamethasone … Look we have these kind of problems…”* (P2)

#### Lack of time

Lack of time and receiving lots of patients at the same time was another reason, which GPs mentioned as barriers regarding patient education and rational prescribing.

*“…Please try to imagine, this is 12 o’clock, midnight, and 15 patients are in the waiting room at the same time. What can I do? I have no time to sit and calmly convince the patient. If I don’t follow their requests and something happens to them afterwards, they blame me and cause problems!…”* (P8)

#### Trainers

All trainers had teaching experience in CME programmes during their professional career. Some of them had been acting as scientific coordinators in CME programmes for several years.

### Theme 1: Robust and clear outcomes

#### Positive attitude towards identified outcomes

Identifying the outcomes of education and defining the expectations of trainees in advance were very positive and useful when preparing the educational programme from the trainers’ point of view. They emphasized the effect of outcome considerations before planning the educational programme, on their attitude and behaviour regarding teaching methods.

*“This programme had a very great effect on my attitude as a teacher regarding the education. I taught in several CME programmes before, but now I notice that we must consider outcomes of education…education should not be transferring series of theoretical issues from books and articles without thinking that who are the trainees and what are their expectations after education.”* (T6)

Outcomes helped them to determine the relevant educational package, suitable teaching methods and assessment. There was a strong belief that OBE is a very good approach for CME because of the complexity of adult learning compared with undergraduate education and the successes of the programme to motivate doctors to stay in the course, participating actively and increasing their competences.

*“During the programme we could evaluate ourselves, we saw that the trainees were following the subject willingly…we didn’t need extra energy to keep them in their seats, (something we are dealing with in other CME programmes), they were asking relevant questions and were taking notes carefully, it was a sign of their learning…after 30 years of teaching at the university, I regret not knowing about OBE before, to use it in my education.”* (T2)

The trainers concluded that the programme would have an effect on participants’ attitudes and performance, based on the discussions during the programme and the feedback, which trainers received at the end of the course. But they also emphasized the continuity of the programme to improve GP’s practice in a sustainable way.

#### Comprehensive content of the programme

From the trainers’ point of view, the content of the programme was not classic medicine subjects. The content included clinical and practical issues and GPs’ “must knows”. Trainers mentioned that they had a clear content and curriculum before starting the education and they built their teaching methods considering them.

*“I can say the content and curriculum of my subject was different than other CME programmes I taught already, and I tried to concentrate on the product of the education -as I learned in the workshop- and think that is exactly what they need.”* (T5)

### Theme 2: Strong adult learning approach

#### Clear educational strategy

The trainers said that they followed different educational strategies and planning compared with other CME programmes. They tried to compile educational outcome oriented plans and design interactive and problem-based teaching methods based on the created framework. Some of them described how they created opportunities for the GPs to challenge their prescribing patterns and change their attitude.

*“We tried to arrange that the discussion be handled by the participants. We encouraged participants to bring up their prescribing errors and we also brought lots of irrational prescriptions to class. Participants were asked to find the irrationalities in the prescriptions and discuss them, so they were involved in the programme and didn’t get tired and were following the course with big interest.”* (T1)

#### Benefits of the teacher training programme

All of the trainers emphasized the influence of the teacher training workshop on their performance from several aspects: creating new views and ideas in their minds; helping them to form an educational framework; designing different teaching methods; and learning useful educational subjects. Some believed that all faculty members should participate in such a workshop and some wanted to take part in another OBE workshop to learn more.

*“In our country, university teachers –especially in medical universities- are specialists in their professions. But regarding teaching they don’t receive structured education. If they are successful, it’s because of their individual characteristics and if they are not, the reason is same. … Even though the OBE workshop was very short, we understood that we must change our methods… In my opinion all faculty members during their professional life must participate in this kind of workshop, not only once, but continuously…”* (T6)

#### Time limitation of the workshop

Despite the fact that trainers found the workshop very useful, they also mentioned that it was too short. They believed that if they had more time to complete the OBE workshop, they could act better in the CME programme. For instance, if the given time was extended, then the workshop could have included more comprehensive material and ensure better understanding of OBE.

*“Sometimes when I am reviewing this programme, I think if we had more time we could perform better. Three days’ workshop was not enough to understand the essence of OBE. We also needed some practices regarding the different educational methods, which we taught during the workshop.”* (T2)

#### Participants’ active involvement

Trainers expressed their satisfaction regarding GPs’ involvement in the educational process and also their satisfaction to participate in the programme. Active listening by participants during the lectures and following the subject, their eagerness to contribute to discussions and asking quite relevant questions, were mentioned by trainers as an interesting part of their teaching experience.

*“One of our big problems in CME programmes is that doctors are not students, they are busy and don’t like to spend any of their work or rest time in classes. They participate in programmes for the CME points, which they need to be re-certified, therefore it is difficult to attract their attention. Participation in this programme was good, maybe we were well prepared… I don’t know, it was possible to see satisfaction in their faces. It was not one-way communication, you could easily see that they were learning…”* (T4)

### Theme 3: Importance of assessment

#### Suitable assessment tools

Trainers appreciated evaluation of the programme results based on designed assessment tools. Knowing what shall be assessed during and after the programme and which suitable tools will be used, before starting to teach was some of the trainers’ first experience.

#### Impact of the programme

Trainers expressed their willingness to receive results of assessments to see if the programme had any effect on GPs’ competences. To be informed about results of the programme was a valuable feedback of education to use in future educational planning according to the participants’ points of view.

Some of the trainers informed the interviewer that they were invited by another organization to conduct same education programme to groups of doctors. They found this action a positive sign of success for the OBE.

#### Barriers for effectiveness of education on behaviour change

Difficulties in changing behaviour were stressed by interviewees despite very well planned educational programmes. The trainers also mentioned some other factors that affected prescribing such as diagnosis, culture of society and economical situation. They believed that some of the doctors are aware of their irrational prescribing, but continue doing it for several reasons. They believed that education about principles of prescription writing must become a subject in undergraduate education before real practice.

*“creating a right behaviour is much better than trying to correct the wrong one”.* (T4)

### Theme 4: Interprofessional teaching

Teamwork was appreciated by the trainers. They described how good it was, when the group of multi-professional trainers took responsibility to train the target group in a friendly concerted action. It was emphasised as a strength of OBE that brought professionals together to design and implement the educational programme.

*“In this programme we built almost everything together with other colleagues. We taught together also…For example I knew what my colleague is going to teach, so I didn’t repeat it…,unlike the other CME programmes which we are invited to teach without any information about the rest of it…”* (T1)

## Discussion

This study demonstrated widespread agreement among both the GPs in the intervention project and their teachers, regarding greater satisfaction with the programme and improvement of knowledge and skills to a higher extent compared with previous CME programmes with traditional teaching methods. The usefulness of the OBE programme was indicated by all interviewees. GPs found the programme related to their needs and expectations. The content of the programme was updated and applicable to their clinical work and the hierarchy of topics had led them to effective learning. Trainers showed their satisfaction to have been introduced to OBE and reported use of this approach in their educational planning, teaching and assessment. The difference between OBE and other conventional CME programmes could be related to the use of constructively aligning [38] the curriculum, with outcomes, content, educational methodology, and assessment building upon each other.

OBE appears to be acceptable to most teachers probably because the concepts of OBE are clear, easily understandable, and provide a robust framework for the curriculum. This approach increased the relevance of the education for the participants’ real practice of medicine [39, 40].

Group discussions had a very important role in the participants’ learning. It was a new experience for them to participate in such an interactive programme since almost all of the traditional CME programmes were mainly teacher-centred and didactic. Furthermore, peer education is an interactive learning method used in medical schools [41] and increasingly recommended in CME [42] to give opportunity for participants to learn from and stimulate each other.

The GPs found the content of the programme to be highly relevant for their professional needs. The trainers’ teaching methods, inter-professional collaboration and structured lesson plans, combined with flexibility regarding questions and discussions made the programme enjoyable for the GPs. The trainers illustrated the relevance of the outcomes to everyday professional life with questions to the participants as well as real examples of irrational prescribing. This approach can be seen as raising the value of the topic in the view of the participants (expectancy- value theory), and perhaps even contribute to increasing their motivation for improvement [38]. Contributing to this was the complementary booklets sent out a month after the end of the programme.

Some GPs reported a change of attitude and performance and a more rational prescribing behaviour after the programme regarding choice and quantity of prescribed drugs as well as quality of prescriptions. This corresponds to the results of the CRCT, where we found changes of attitudes and skills [31], as well as significant improvement of prescribing performance after the OBE programme, confirmed by evaluation of 13,480 prescriptions by the participating GPs [32].

The importance of patient education to reduce irrational requests was raised by the GPs during the interviews. What is rational in a medical sense may perhaps not look appropriate for the patients [29], so patients’ awareness of correct treatment and risks with irrational use of medicines need to be enhanced. Several interviewees mentioned the value of mass media in terms of patient education, which can be one way of achieving a more consensual view on rational use of medicines between patients and doctors [43].

Doctors’ prescribing is also influenced by socio-cultural and socio-economical factors [29], such as patient demand and pressure to follow their requests for keeping them as clients, workload, number of patients and time pressure [44]. Based on our results we can add feeling insecurity, because of intimidation by their patients and their patients’ family members. Violence or threat of violence against physicians and residents is an important issue from professional and social aspects, which needs to be considered for the development of effective and preventive interventions [45, 46].

Pharmacists’ selling drugs without prescription, despite being illegal in Iran, was stressed by GPs as a major problem in rational drug use. They believed that pharmacists followed the patients’ irrational requests, which may lead to serious problems. Buying medicine without a prescription signed by a medical doctor from community pharmacies despite being illegal is a major health challenge in several countries and is one of the reasons for the increase in the antibiotic resistance [47–49].

Trainers appreciated the inter-professional approach of this programme. They were able to answer participants’ questions and carry out discussions together as well as determining course plans and presentations. This may be one of the reasons for the increased effectiveness of the OBE. Inter-professional education is challenging, but by demonstrating respect for other professionals, communicating in a clear and effective way, following common goals [50, 51] and using appropriate teaching and learning approaches, it is possible [52].

### Methodological considerations

Three issues of trustworthiness should be considered in order to evaluate and generate the findings: credibility, dependability and transferability [34, 53]. Credibility and dependability were enhanced through a thorough analysis, where one co-author separately coded some of the interviews and the other co-authors took part in discussion on the emerging codes, sub- themes and themes until consensus was reached. Furthermore, the participants and some experienced colleagues outside the research team were invited to give comments on the results of the analysis. In addition, the broad composition of national and international researchers in the team brought different perspectives to the analytical and interpretative process.

There is a possibility of social desirability bias in the responses from the participating GPs, as the interviews were conducted by the coordinator of the CME training. However, the coordinator was not directly involved in the training sessions, and more importantly, the GPs were always asked to give concrete examples of their statements, not just general comments. A similar bias could be expected for the interviews with trainers, but was avoided as those interviews developed as reflective discussions, with lots of concrete examples from the training sessions. This may have been facilitated by the fact that the interviewer herself had a background in medical education.

This study aimed at exploring the perceptions of the trainers and GPs after participation in this particular CME programme and cannot be generalized as findings in themselves. However, as this is one of very few examples of how participation in an outcome-based CME programme is experienced by participants, we believe that our findings can be useful for both educational planners and physicians at an international level.

## Conclusions

Our findings support the notion of usefulness of OBE to improve level of competence for both GPs and CME trainers. We are therefore suggesting OBE as a promising approach when creating CME programmes for physicians in Iran, and that similar approaches could be considered in other contexts, both regionally and globally.

## Abbreviations

CRCT: Cluster randomized controlled trial
CME: Continuing medical education
GPs: General physicians in primary care
OBE: Outcome-based education.
